# The Making and Fitting of Spectacles and Eyeglasses from the Optician’s Standpoint1Read before the Section on Ophthalmology, Otology, Rhinology and Laryngology, Buffalo Academy of Medicine, March 18, 1901.

**Published:** 1901-06

**Authors:** P. H. Meyer

**Affiliations:** Buffalo, N. Y.


### THE MAKING AND FITTING OF SPECTACLES AND EYEGLASSES FROM THE OPTICIAN'S STANDPOINT.[1]

BY P. H. MEYER, Buffalo, N. Y.

THE title, An Optician, suggests to me that it is one which covers a broad field of labor and science, compared with that which it occupied a generation ago. In line with the many discoveries in science, and the great improvements in mechanics, the work of the optician has been divided into many branches, each of which in a comparatively short space of time has developed into practically a science of its own. Among these the ones in which the physician is most actively interested no doubt are microscopy and refraction.

The microscope is the product of the labors of the physician and optician, as also are the ophthalmoscope and spectacle, as they are made today. They were planned and demanded by medical science and urged by the great strides in that study, they were brought to their present state of perfection.

To a person who has given the subject no thought it is probably difficult to realise wherein any great amount of science and mechanical ability are involved in the production of a pair of spectacles. It might be well to explain, at this point, that I do not refer to the glasses manufactured in large quantities and sold over the counters of dry goods and jewelry stores, to people with presbyopia and myopia, many of whom really require the attention of an oculist, and

---

1. Read before the Section on Ophthalmology, Otology, Rhinology and Laryngology, Buffalo Academy of Medicine, March 18, 1901.

in an endeavor to avoid the supposed unnecessary expense of that course accumulate a small stock of spectacles.

Many people, if we follow from the beginning of their experience with the wearing of glasses, do so with the idea that a pair of spectacles are what they need to give them relief from headaches, nervousness or some ailment with the eyes themselves. They accordingly visit some store where glasses are sold, and where perhaps a professional looking man will seriously look at their eyes through mysterious looking instruments, and they are subjected to what appears to them a very scientific examination. Of course, they buy a pair of glasses and are confident at the time that they are going to be forever relieved from their ailments. These impressions, however, do not last long, as the course they have taken in the majority of cases of this kind, has a tendency to almost as abruptly lead them to a physician, as though some one had grasped them by the ear and led them to the door of the doctor.

This assertion is proven by the fact that in the course of ten years' observation from the position of a prescription-filling optician, I find that a large percentage of the people from whom I receive prescriptions have in their possession one or several pairs of glasses, which they had purchased from some one and which were not prescribed by a physician. The harm resulting from this course, in the majority of cases, is one of the main roads that lead the subject to the place where medical science is involved in producing spectacles and eyeglasses. Another is by way of the general physician to the eye specialist, and a third that of the patient directly to the oculist.

Prescription in hand they visit the optician. I want to say here that at this stage the most essential part of suiting the patient with glasses is completed. But the question is, do most people, and I might even say physicians and oculists, realise the importance of the correct filling of that prescription, not only in relation to the perfect grinding of the lenses as prescribed, but also to the making of the frame, and adjusting them to the correct position before the eyes?

Here we have a branch of the optical business in which expert mechanism, precision and accuracy can be exercised with much farther reaching results than most persons realise.

Consider that a pair of glasses are carried on the face, that part of us which we most thoroughly shield from unbecoming additions, and that they are worn in connection with that most delicately constructed organ, the eye. Few persons, if any, would wear glasses from choice; if so, no doubt a short period of experimenting would suffice to alter their opinions, especially if they were not perfectly fitted. Glasses on most anyone can be worn with absolute comfort, as they can be so adjusted as to leave the wearer entirely unconscious of them after a comparatively short space of time is allowed, in which to become accustomed to them. Furthermore, when they really conform to and fit the face, they are not detrimental, but in many cases improve the appearance of the wearer.

Probably no one realises as thoroughly as the oculist the importance attached to the position in which lenses are worn before the eyes. When we consider that the nature of the curvature and shapes of lenses are limited to a few—namely, spherical and cylindrical, convex and concave and prismatic, it seems a simple matter to mount and set them on the face. On the other hand, if a prism representing one degree of a circle correctly prescribed, will give a patient relief from headaches, stomach or nervous troubles, will not a prism of similar extent produced by incorrectly adjusted lenses cause the same trouble? And if a difference of five degrees in its axis in a slight cylindrical curved lens will relieve or distress the patient, does this not illustrate the importance of the lenses being set in a correct position?

We all know that no two faces are exactly alike, nor are two noses, consequently no two pairs of glasses dare be alike, if they fit perfectly. Does this not represent a field in itself for the practice of accuracy in mechanics?

Recently a nearsighted man said to me, "A few years ago I used to go into a store and fit myself out of a tray of glasses and now since doctors and opticians do my fitting, I am entirely dependent upon them for comfort." Upon asking him if he would like to go back to the old method he said, "no indeed, not I." That same gentleman visits the optician not less than once a month to have his glasses adjusted.

As an example, allow me to follow him a little farther; he has an inclination to hold his head a trifle higher than most persons, which fact was overlooked in the fitting of his glasses. This, of course, gave him a tendancy to look a trifle below the center of his lenses most of the time. He complained of headaches and his stomach. The lenses were lowered 1-16 of an inch before his eyes and he was thereby relieved. The change in the position of the lenses obviated the effect of one degree of prism produced by the optical center of his lenses, being set a little higher than the visual plane.

Another case of the importance of accuracy in adjusting lenses before the eyes, is shown in one of a lady who recently traveled one hundred miles to her oculist. She was suffering with headache and her stomach. The prescription which relieved her was this, "reduce the angle of glasses five degrees." The fact that her glasses tilted a few degrees more than they should, no doubt gave the effect of a cylinder in a horizontal meridian, producing an astigmatic effect which she did not naturally have.

In a work on spectaces and eyeglasses by Dr. Phillips, a table is given wherein is shown the additional extent of cylindrical and spherical effects caused by incorrectly adjusted lenses, as cited in the above case.

Any optician engaged in making glasses according to prescriptions, could no doubt cite many cases wherein a difference of a small fraction of an inch in the position of the optical center of lenses before the eyes had given considerable discomfort. Of course there are many wearing glasses, who are not affected by a slight error in adjustment of the lenses, but I find that nearly all and especially those with a stomach or nerve ailment, are hypersensitive in regard to the position of their lenses.

It is a common occurrence to have a busy man or woman, whose glasses have become bent out of shape, leave their work during the rush of business to step into the store of an optician to have them readjusted, and in most cases they are out of line not more than 1-16 or 1-8 of an inch, which is sufficient to make most persons wearing glasses uncomfortable. As a rule, the optician does not charge for this service, which to me is a source of gratification because of these reasons: first, as it is a simple matter for the optician, requiring no further outlay than a moment of his time in its performance; second, for the slight service one is fully recompensed when the person in words and facial expression testifies to instant relief.

This is especially noticeable in the small boy who wears strong lenses, and who has had his frame bent out of its correct shape in a scramble. After readjusting his glasses, to hear him say, "Gee, that's fine." It is an easy matter to feel that he has some change coming to him.

To make glasses so that their frames do not easily bend out of shape and still be delicate in appearance, has been a subject of much study and experimenting on the part of the optician. No metal or other material has proven nearly so satisfactory for this purpose as gold, which after all is the most appropriate to wear on the face. In mechanical manipulation any mechanic of experience in this respect will grant its superiority. It is most cleanly, as it does not tarnish, rust or accumulate verdigris, as do metals sometimes used. It can be most satisfactorily repaired, and as it outlasts several of those made of any other metal used for this purpose, it is most economical.

Next in order to the correct fitting of a pair of glasses, is that they should remain so for as long a time as possible. That they be well made is, of course, essential to their being a perfect fit. The methods of writing measurements of the curvature and axis of lenses, so as to be legible to the physician and optician, have long been standardised. It is only within the last few years that the mountings of glasses have been produced in their present state of mechanical perfection, both in regard to the manner of fitting them before the eyes and the division of the material, so as to give the best results in their use.

Spectacles will probably always be considered the most satisfactory form of mounting lenses, mainly because of their remaining in a more stationary position before the eyes than do nose glasses. The latter style, while greatly improved in its construction during the past few years, is still the same in principle of adherence.

The eye glass for dress is neatest in appearance and most convenient for momentary use, but the spectacle will always be most practical, both in regard to its scientific value and the comfort derived from judicious division of its weight, in adjusting to the nose and ears.

Lens grinding is a branch of spectacle-making in which machinery is made to play an important part; not only is it conducive to labor-saving, but it is also valuable because of its regularity of purpose. However, no machine has been made that is its own master and judge of its work. It is necessary for the prescription optician to employ at least one expert lens-grinder, who in his capacity requires to be an artist in his line, if good results are to be had. To produce a lens with perfect regularity of curvature and entirely free from opacity requires skill and an eye trained by long experience. Since so many lenses are required wherein the optical center is in a different position from that of the geometrical one, as in lenses combined with prisms, the expert lens grinder has grown considerably more necessary than heretofore, where glasses are made according to prescription.

It would require a visit to the optician's workshop to realise the mechanical skill and accuracy exercised in the completing of a well made pair of glasses. Aside from the importance of precision and care necessary in the making of an instrument to be used in connection with the human eye, it is a difficult feature of workmanship to combine metal and glass when neatness and delicacy are required. Everything is made to correspond with measurements, the patient choosing the style of glasses, their face is measured and each part made to conform to the peculiarities of the wearer. Individualism is necessary in perfect fitting glasses. We all have our own faces, so must we all have our own spectacles to be entirely comfortable in using them.

The refractionist well knows the many different grades of lenses, curvatures and combinations that the optician is required to make. While there are not very many kinds of glasses, if we consider their general style only, exact similarity in completed pairs, when actually fitted to the face, are far less common than is sameness in the prescriptions after which the lenses are made.

It is true that only a few years ago when glasses encircled with a rim were most generally used, the sizes of the lens rims were limited to less than half a dozen, but since the event of rimless glasses, there is no limitation to the sizes and shapes lenses are made in their adaption to the face. Even in rimmed glasses, which at this time are used only where durability is necessary, as in cases of small children, there is a considerably larger variety of sizes than were used in former years.

A feature especially noteworthy in our branch of spectacle-making is a class of people who form a majority of those who are our patrons. The fact that errors of refraction in the eyes and muscular irregularities are frequently the cause, or are contributory to the nervous and stomach ailments and headaches, will suggest to you that persons afflicted with this class of difficulties are the most numerous of our patrons.

As to the relation of our work to that of the physician, in comparison, our business and that of the druggist are more nearly similar than any other mercantile line. It, however, greatly differs in that we deal directly with the principle, while the druggist successfully fills prescriptions without a visit from the patient.

Of course, spectacle-making is a mechanical vocation in every sense, but spectacle-selling is not, and in this respect I do not know just what mercantile line of business with which to compare the optician's work. I think almost every merchant could recall instances of dealing with a customer, who would not be suited, regardless of every thing that he could do for them. As they leave his store he will content himself with the thought that the person was a crank.

I by no means believe the optician's customers to be cranks in the sense with which the dictionary defines the word, but I do believe that in general they are a different class from those with which other merchants do business.

If we could imbibe into a model storekeeper a thorough knowledge of spectacle-fitting without that of spectacle-selling, and place him suddenly in an optician's store, he would probably believe that most of his customers where whimsical. By this I mean to illustrate that the spectacle-making optician requires, in order to be successful, a certain different sort of mercantile training from any other class of storekeeper, which only years of experience in this branch of business will develop.

Fortunately for the optician, some oculists instruct their patients in some cases as to the style of glasses he wishes them to wear. Especially when spectacles are to be worn when nose glasses are preferred by the patient is this fortunate, as it is a difficult matter to make such persons understand that spectacles are what they ought to wear. Between himself and the patient the physician undoubtedly is master of the situation while relatively the optician is not always.

There are quite a variety of styles and shapes of glasses made, and as in any article of this nature only one kind is the best suited to each case. The optician is expected to know which is that best kind and if it does not at the time suit the cosmetic taste of the patient, the well-trained optician will overcome the situation by the customers' having that which is best for them and, of course, with perfect work always resulting in the retention of the person's good-will. There is no class of patrons that a first-class optician would rather deal with than those who have previously been ill-fitted. No one except those who wear glasses can realise what a continuous source of discomfort an incorrectly fitted pair of glasses are. I believe most persons perfectly fitted with glasses do not forget the optician who made them. This fact coupled with the appreciation of the successful oculist, of perfection in this class of work, constitutes the foundation upon which a spectacle-business is established.

I noticed recently in a local Sunday paper an article boldly headed "King of Oculists." It described one of fame in this branch of medical science in Europe. Among others noted, one of the most distinguishing features of this physician's work, was that he always insisted on his patients going to one certain optician in his city for their glasses, and much as was his confidence in this man's ability he always had the patient return with the glasses to him, so that he could be sure that they were perfectly made and adjusted. As exactly the same course of procedure is insisted upon not only by the far-famed oculists of our city but also by some of the locally famous ones, to make this fact noteworthy in our country in the description of a European oculist seems to me far fetched. I, for one, am in a position to know what a superior nature of workmanship in glasses some oculists of Buffalo require in order to bestow their approval.

I believe that no one realises more thoroughly than the leading oculists of our city, how essential a perfectly made and adjusted pair of glasses are to success in the results of their use. The perfect fit is not usually obtained by a haphazard manner of buying nor making them, but by judicious choosing of an optician who will make and fit them according to a plan based on scientific truths—a plan in the following of which the conforming them to the individuality of the face and needs of the wearer has been the main consideration.

A great deal can be said in describing the making of glasses, not only in regard to the grinding of the lenses from the raw material to the completing of the spectacles, and more especially of the mechanical ability required in making the final adjustment in correctly fitting them to the face. But as the physician cannot be much interested in purely mechanical procedures, I have endeavored to remain in the province including the consideration necessary to the successful making and adjusting of glasses. That these are numerous you will grant from your own experiences in your profession, wherein great depth and technicality are involved in everything pertaining to it.

Since the medical profession have interested themselves in spectacles these conditions have at least, in a small degree, blended themselves into the art of spectacle-making. They are an article of which the physician is the architect, and you surely will appreciate that if their correct prescribing requires long years of study in addition to the great problem of medicine, they at least require mechanism of precision in their making. If not I want to say that much of the energy of my business life has been wasted.

THE GENESEE, 532 MAIN STREET.

